# High level of vaccination and protection against hepatitis B with low rate of HCV infection markers among hospital health care personnel in north of Iran: a cross-sectional study

**DOI:** 10.1186/s12889-020-09032-6

**Published:** 2020-06-12

**Authors:** Saffar Hiva, Khoshayand Negar, Parsaei Mohammad-Reza, Ghorbani Gholam-Reza, Aarabi Mohsen, Nadi Ghara Ali-Asghar, Saffar Mohammed-Jafar

**Affiliations:** 1 Department of Pathology, Shariati Hospital, Teheran University of Medical Sciences, Tehran, Iran; 2 Resident of Pathology, Shariati Hospital, Teheran University of Medical Sciences, Tehran, Iran; 3 Mazandaran University of Medical Sciences, Sari, Iran; 4 Health Sciences Research Center, Addiction Institute, Mazandaran University of Medical Sciences, Sari, Iran; 5 Research Center for Pediatric Infectious Diseases, Department of Pediatric Infectious Diseases, Bu-Ali Sina Hospital, Mazandaran University of Medical Sciences, Pasdaran Bolv, Sari, Iran

**Keywords:** Health care personnel, Hepatitis B virus, Hepatitis C virus, HBV vaccination coverage, HBV booster

## Abstract

**Background:**

hepatitis B virus (HBV) and C virus (HCV) are among the leading causes of mortality worldwide. Health care personnel (HCP) are subjected to increased risk of these infections. Therefore, HBV vaccination and post-vaccination serologic testing (PVST) are recommended for them. Our objectives in this study were investigate how well the vaccination guidelines for hospital HCP_s_ were implemented. Moreover, the prevalence rates of HBV and HCV infections were calculated. To determine the presence of immunological memory, vaccinated personnel negative to antibody against HB surface antigen with one dose of HB vaccine were boosted.

**Methods:**

From 1 July to 30 November 2017, a cross-sectional study among HCPs working in public hospitals were conducted. All HCPs from various professional categories potentially at risk of exposure to contaminated sources were included. The information was gathered via interview and self-administered questionnaire. The questions were focused on the demographic characteristics, HB vaccination and immunity status and time elapsed since initial vaccination series, and frequency of needelstick injuries during the past 12 months of their work. Moreover, the prevalence rate of HBV and HCV infections were calculated. To determine the presence of immunological memory, subjects negative to HBV seromarkers received a booster dose of the vaccine.

**Results:**

A total of 186 out of 766 participants were male and nurses comprised 71% of personnel. Although all HCP were vaccinated, 84% of them completed the course and less than 5% of them received PVST. According to the results, 0.78, 4.6, and 83% were serologically positive to HBV surface antigen, antibodies against HBV core, and S antigens, respectively. Approximately, 91% of seronegative participants responded to a booster dose and only 0.91% of the personnel was anti-HCV positive.

**Conclusion:**

Most HCP received full HBV vaccination course. Although a minority did PVST, the HBV vaccine-induced long-term protection and HB vaccine booster were not required. Therefore, policies should be made to increase the rate PVST after immunization. According to the results, the HCV infection rate was low and thus pre-recruitment screening was not necessary.

## Background

Chronic hepatitis B and C infections are among the major public health issues and also among the leading causes of liver-related diseases and mortality in the world [1, 2].

According to the World Health Organization’s (WHO) estimation, approximately 257 million persons are living with chronic hepatitis B (HBV), which led to more than 887,000 deaths in 2015 [3]. In addition, studies found globally nearly 122 to 185 million people are anti-HCV antibody positive [4] WHO estimated that in year 2016, approximately 399,000 people died from HCV related illnesses [5]. After childhood, the main routes of transmission are percutaneous sharp injuries, sexual and mucosal exposure to infected blood/body fluids (B/BF) [6–8].

Health Care Personnel (HCPs) are typically exposed to injuries by sharp instruments in the course of their duty. Injuries by sharp objects and related risk of infections represent the major risks related to occupational health and safety of HCPs worldwide [9–11]. According to data provided by the WHO, there are approximately 36 million HCPs worldwide, of whom around 3 million/ year receive an injuries with an occupational instruments with nearly 2 million exposure to HBV and 1 million to HCV [12]. The limited available information about sharp injuries causing transmission of HBV, HCV and HIV indicated 0.42 HBV infection, 0.05–1.3 HCV infection and 0.04–0.32 HIV infection per 100 sharp injuries [13]. HBV is a vaccine-preventable disease [6–8] and HCV is a treatable infection [5].

A range of measures and interventions can be used to minimize the transmission rates among HCPs [9–11]. However, immunization of HCP against the risk of HBV is considered to be an additional most effective strategy for prevention and protection from HBV infection [6–11]. In this regard, several international agencies [14, 15] and advisory committees on immunization practices (ACIP) in different countries [16, 17], including Iran [14], recommended that all HCPs at risk of exposure to B/BF should be vaccinated against HBV infection. Moreover, guidelines suggest that vaccinated HCP should verify their immunity status within 1–2 months after completion of the course [5–7, 14, 16, 17]. Despite these recommendations and effective protection profile provided by immunization among vaccinated personnel, international studies have reported suboptimal and varying vaccination coverage rates among HCP in different countries and even among various subgroups within the same country. For example, the reported rates of complete HBV vaccination was 24.7% in Africa [15], 63.4% in US [18], and 85–100% in EU countries including Poland [19, 20]. In addition, results of studies on the immunogenicity of HBV vaccine among adults, including HCP, indicated 10–20% non-responsiveness among vaccinated adults [6–8, 21]. In the case of exposure, having a good knowledge of HCP immunity status is important for appropriate post-exposure management. Unknown status is a cause of anxiety in both personnel and health professionals responsible for their management. This may result in hasty decisions regarding prophylactic measure and unnecessary use of HBV immunoglobulin (HBig) [16]. Despite these recommendations, a minority of vaccinated personnel documented their post-immunization status.

This study was designed to determine how well the guidelines for HBV immunization of HCP had been implemented. Moreover, the point prevalence of HBV infection seromarkers and HCV seroprevalence among different subgroups of HCP working in hospitals was measured. Moreover, to assess the durability of vaccine-induced immunity and protection, immunologic responses to an HB vaccine booster dose administration in personnel seronegative to all HBV infection seromarkers was evaluated.

## Methods

This descriptive-analytical cross-sectional study was conducted from 1-July to 30-November 2017, to investigate the HB immunization status and post- vaccination serological testing (PVST), HB infection seromarkers, and HCV infection prevalence among HCP working in the public hospitals in the western areas of Mazandaran Province in North of Iran. The west of Mazandaran Province North of Iran, consist of 8 main districts. In each main district, one public hospital responsible for medical services to inhabitant exist. For this study purpose 4 out of 8 hospitals were randomly selected. In the selected hospital all eligible HCP from various professional categories deemed as the representative of all HCPs working in the region were recruited to participate in this study. In each hospital, selection and recruitment of eligible personnel was supervised by hospital nurses responsible for infection control and prevention associated with one of the author.

An HCP is defined as a person from the following category, who has professional contact with patients and/or is potentially at risk of exposure to contaminated sources inside hospitals: physicians, nurses and nursing aids, midwives, laboratory staff, and cleaners (logistic personnel involved in the waste disposal).

The information collected from HCP was done via interview and a self-administered authors made questionnaire (presented an additional file No 1), The questions focused on the age, sex, marital status, history of major surgery duration of work, and/or blood transfusion, IVDU and also on the primary vaccination status and the number of vaccine doses received, PVST status, time elapsed since the primary vaccination, and possible receipt of additional doses of HB vaccine and/or Hepatitis B immune globulin (HBIg) and the possible frequency of sharp instruments incident in the past 12 months of their work were sought. The research population was comprised of healthy HCP working in hospital. Those with serious systemic or metabolic disorders, malignancies or immune deficiencies, and those who received blood/plasma within the past 6 months were excluded. The study protocol used the standard ethical guidelines and was approved by the Ethics Committees of Mazandaran University of Medical Sciences (Sari: 3087-JR, Mazums, Rec. 1390.3087) and Tehran University of Medical Sciences (318563–37,970–30-01-97). After obtaining informed written consent, and filling an anonymous questionnaire 5 ml of venous blood of all participants was collected and stored at − 20 °C to measure HBV serological markers: [HBV surface antigen (HBsAg), antibodies against HBsAg (Anti-HBs) and against HBV core antigen (Anti-HBc)]. The seromarker concentration was measured using ELISA according to the manufacturer’s instructions (Quantitative Anti-HBs, HBsAg conversion ULTRA, qualitative Anti-HBC, all kits from DIA; PRO. Diagnostic Bioprobes Srl, Miano–Italy). The in-vitro diagnosis of HCV infection was done using a third-generation ELISA, which was sensitive for the detection of IgG-class antibodies against the viral structural protein (HCV-A6, 3rd generation immunoassay, DIA.PRO). Finally, the equivocal test results were rechecked.

Full vaccination was defined as receiving three doses of HBV vaccine. The vaccinated individuals with anti-HBs titers > 10 IU/L were considered immune; whereas, vaccinated HCPs with positive titers of both anti-HBs and anti-HBc were regarded as having immune derived from natural HB infection (breakthrough infection). Cases with isolated anti-HBc positivity without HBsAg were considered possibly infected to HBV and followed accordingly. Positive results for HBs Ag were considered as breakthrough infection and possible vaccine failure. Anti-HBs antibodies titer less than 10 were considered to be nonseroprotected and offered to receive one booster dose. Four to 6 weeks after booster injection, anti-HBs concentration measured. Seroconversion was defined as the presence of immune memory. Persons with isolated anti-HBs titers > 250 IU/L vaccinated for more than 10 years earlier without receiving any additional dose of HB vaccine or HBig was arbitrarily considered to be having immune by natural booster. All participants positive to anti-HCV antibody were retested and followed appropriately. Those positive for isolated anti-HBc and subjects positive for anti-HCV antibody were examined by PCR methods. (Ampli SensR HCV-FRT, A analytical sensitivity, and Ampli SensR HBV-FRT, A analytical sensitivity).

### Statistical analysis

Collected data was analyzed using SPSS version16.0. The descriptive statistical method was used in the form of mean and standard deviation (SD) for age, duration of work, immunization at the confidence interval of 95% (CI = 95%). In addition, the frequency of variables among studied HCP was used to calculate collected data. The Chi-Square and Student t-test were used to find differences between variables. Results were considered to be statistically significant when the *p*-value was less than 0.05.

## Results

Among 914 eligible HCP, 776 HCP (83.80%) consented to participate in the study. Of them, 186 (24.28%) were male and 580 (75.72%) were female. The number of cities population, eligible and participated hospital HCPs, with their demographic characteristics, mean age and mean duration of working in Table 1, relative contribution of various professions distribution of each job categories participated in this study in Table 2 and vaccination status, HBV and HCV infection seromarkers in Table 3 are presented. Approximately 73.5% of personnel were married, one person without any evidence of infection was IVDU. Overall, 42.4% of HPCs experienced ≥1 needelstick injuries in their last 12 months of work.

**Table 1:** The number of cities population, eligible and participated hospital HCPs, with their Demographic characteristic based on their own district in the west of Mazandaran province, North of Iran

Districts	Districts× 10^3^ population	Eligible HCP	Participated HCP (%)	Mean age year /range	Mean duration of work year /range	Male/ Female
Nour	195	187	154 (82.3%)	33.47/22–59	13.22/1–26	41/113
Chalus	220	316	235 (74.3%)	33.85/22–58	12.93/1–27	64/171
Tonekabon	185	175	148 (84.5%)	34.59/22–59	12.76/1–25	32/116
Ramsar	110	236	229 (97%)	34.06/22–60	12.17/1–30	49/180
Total	710	914	766	33.75/22–60	12.77/1–30	186/580

**Table 2:** Relative Contribution of various professions distribution of different job categories participated in the study according to their own hospital in the west of Mazandaran province, North of Iran

Districts	Professional categories number					
	Nurses	Midwives	Laboratory technicians	Physicians	Cleaner	total
Nour	113	10	19	9	3	154
Chalus	165	19	32	14	5	235
Tonekabon	105	9	21	10	3	148
Ramsar	164	17	29	13	6	229
Total	547	55	101	46	17	766

**Table 3:** Profiles of HB vaccination status, HBV and HCV infections seromarkers among different professions of hospital HCPs in Mazandaran province, North of Iran

Job categories total number	Vaccination Status		Hepatitis Seromarkers				
	Full n = 644 (84%)	Incomplete n = 122 (15.9%)	anti-HBs n = 636 (83%)	anti-HBc n = 36 (4.69%)	HBsAg n = 6 (0.78%)	Natural boosting n = 144 (18.79%)	Anti-HCV n = 7 (0.91%)
Nurses n = 547 (71.4%)	461 (84.72%)	86 (15.72%)	466 (85.19%)	14 (2.55%)	3 (0.54%)	99 (18.09%)	4 (0.73%)
Laboratory staff n = 101 (13.1%)	84 (83.16%)	17 (16.8%)	81 (80%)	9 (8.9%)	2 (1.98%)	23 (22.77%)	2 (1.98%)
Midwives n = 55 (7.1%)	46 (83.63%)	9 (16.3%)	41 (74.54%)	6 (10.6%)	0	11 (20%)	0
Physicians n = 46 (6.0%)	42 (91.5%)	4 (8.7%)	38 (82.6%)	4 (8.6%)	1 (2.1%)	6 (13%)	0
Cleaners n = 17 (2.2%)	11 (64.7%)	6 (35.3%)	10 (58.8%)	3 (17.64%)	0	5 (29.4%)	1 (5.85%)

HB vaccination and infection status: all HCP participated in this study were vaccinated against HB. However, 122 (15.92%) participants did not complete the vaccination course, that 48 and 74 of them received one and two doses, respectively. Of all vaccinated personnel, only 37 (4.83%) received anti-HBs antibody serological testing on schedule. However, 155 out of 766 (20.23%) HCP received serological anti-HBs testing because of needlestick injuries during their work’s experience. Of them, 93 subjects (60%) with one dose of HB vaccine were boosted. A total of 6 out of 766 subjects were HBsAg positive: 0.78% (95%CI: − 0.104-1.698); in addition, 36 out of 766 were ant-HBc positive: 4.69% (95%CI: 2.57–6.69), among whom 32 cases were also ant-HBs positive (subclinical resolved HB infection). None of the participants with isolated anti-HBc was HBV-DNA PCR positive. There was not any correlation between the age, and duration of work, with HBsAg and/or anti-HBc positive rates (P = 0.850). From 766 screened HCPs, 636 (83%) (95% CI: 81.2–86.7) showed isolated anti-HBs antibody titers of more than 10 IU/L (seroprotected) and 88 (11.48%) (95% CI: 8.8–12.3%) were susceptible to HB infection. There was no significant difference between fully vaccinated and incomplete vaccination and the gender (df:1, for both variable, P:NS), these data all presented in Table 4. In this study the point prevalence of natural boosting was 144/766 (18.79%). These data are presented in Table 3.

**Table 4:** Profiles of immunity of hospital HCPs in relation to their vaccination status and gender^a^, Mazandaran province, North of Iran

	Vaccination Status^b^			
	Full n = 612		incomplete vaccination n = 112	
	Immune n = 539	susceptible n = 73	Immune n = 97	susceptible n = 15
females	403	57	77	12
males	136	16	20	3
total	539	73	97	15

^a^: Statistical analysis: df = 1 P for both, Non-significant

^b^: Of 766 studied HCPs, 42 were naturally infected the remainder 724 were included

### Boosting study

In this study, out of 88 personnel negative protective titers of anti-HBs, 78 including 4 cases with isolated anti-HBc agreed to participate in the boosting study. Four to 6 weeks after the first dose of HB vaccine booster administration, 71 (91%) participants including all 4 subjects positive for anti-HBc responded to boosting and seroconverted. (Response rate: 67 of 74 (90.5% Vs 4 of 4 (100%).

### HCV antibody test

Among 766 studied HCP, 7 (0.91%) cases (95% CI: 0.33–1.79) were positive to HCV antibody. Of those 5 cases consented to participate and were tested by HCV-RNA PCR. According to the results, none of them was positive.

## Discussion

Study showed that all participants were immunized against HB, but nearly 16% of them did not complete their vaccination course. However, a minority of vaccinees performed PVST. Most of studied HCPs were young in their age and work. Based on our data, 83% of studied subjects were serological immune to HBV infection, 0.78 and 4.6% showed evidence of chronic and resolved HB infection, respectively. Although 12% of vaccinated personnel were serologically susceptible to HBV, most of them retained their specific immune memory demonstrated via response to a HB vaccine booster dose injection. The prevalence rate of anti-HCV was low. Finally, study results indicated that more than 42.4% of HCPs experience at last one NSIs in their work within the preceding year.

Based on this study findings most studied personnel were fully vaccinated. This proportion is one of the highest rates of vaccination coverage among HCP with the reported prevalence in Iran [22, 23] and other countries [15, 18–20, 24–27]. The reported rates of HBV immunization in HCP from both developed and developing countries varied widely [15, 18–20, 24–27]. Whereas, only 11.4% of Cameroonian HCP were fully vaccinated against HB [26], nearly all surgical nurses in Polish hospitals were immunized [20]. The self-reported rates of complete vaccination detected in national survey was 63.4% in the United States of America [18], 55.4% in India [24], and 60% in China [25]. However, this rate was 85–100% among health care providers working in different European Union countries [19, 20]. Similar to our findings, during recent years relatively high rates of immunization against HB in HCPs working in hospitals, from different regions of Iran were reported [22, 23]. In this regard, to estimate the HB vaccination coverage rates among Iranian HCP two systematic reviews and meta-analysis were conducted [22, 23]. In the first study [22], 6311 subjects from 21 articles were included. The history and the complete vaccination rates were estimated to be 86.9 and 70.3%, respectively [22]. The second one, performed among 4104 Iranian physicians and nurses, showed that the history and full HB vaccination rates were 88.7, 93.5 and 73.1%, 76%, respectively [23]. The most possible explanation for the higher rates of HB vaccination coverage in Iran may be due to easy access to free HB vaccine in the hospitals across the country. In addition, the important roles of the occupational health promotion and infection prevention/control committee established in most hospitals should also be considered.

Our data showed that only a minority of surveyed subjects (< 5%) checked their anti-HBs titers on the recommended schedule. The probable reasons for this rate could be explained as follow: 1) The employees should be charged for PVST, which is a rather expensive test, 2) Many of them believe that vaccination is associated with full immunity, so they feel no requirement for PVST. Actually it appears that most HCPs do not have enough or accurate information about PVST. However, during their work’s experience, nearly 20% personnel did anti-Hbs serological testing as part of their post-exposure management. After HBV vaccination, serological testing to document immune response is recommended for persons whose post-exposure clinical management depends on the knowledge of their immunity status. However, the global rates of post-vaccination serotesting varied greatly and the majority of the vaccinated HCP did not check their anti-HBs levels after vaccination [24, 27–30]. Despite applying PVST in the vast majority of EU countries, serologic testing rates were markedly different among these countries [19]. For example, data from a large teaching hospital in France showed that 65% of vaccinated personnel tested their anti-HBs antibody on completion of immunization [19]; whereas, this rate in a multicenter study in Poland was 24.9% [30]. Similarly, in a study on medical students from São Paulo in Brazil, out of 675 participants, 48.9% were fully vaccinated and only 34.8% of vaccinated subjects performed antibody testing [27]. Based on our data and the literature results, particular attention should be given to post-vaccination antibody testing to ensure that vaccinated personnel at risk have developed adequate immunity against HB infection. Therefore, it seems reasonable that testing of antibody on completion of vaccination should be incorporated into a vaccination program on a mandatory basis.

The results from several long-term follow-up studies on the HB vaccinated people indicated that the vaccine-induced antibody concentration declined with time and could reach to non-protective or even undetectable titer without further booster injection or subclinical breakthrough HB infection, 15–23 years after the initial immunization [31–33]. Based on our surveyy, protective antibody titers remained in 83% of the screened participants many years after the primary course of HB vaccination. However, study showed that 93 out of 766 (12.0%) of HCP were boosted with ≥1 dose of HB vaccine during their professional career. This high-rate of seroprotection may be due to the younger age of personnel (mean age 33, years), shorter time elapsed since the primary course of vaccination (46.5% of them had work’s experience of fewer than 10 years), furthermore 12.0% received an additional dose of HB vaccine, occurrence of subclinical breakthrough infection in 4.6% of them, and development of natural boosting in nearly 19% of subjects during their career).

Based on our arbitrary definition for this study, 144 persons showed evidence of natural boosting during their profession. The prevalence of natural boosting varied in different settings across the world, obtained from long-term prospective studies. In a 20-year follow-up study on immunized children born from HBsAg positive mothers, the reported rate of natural boosting was 5.93% [31]. The rates reported in a 22-year prospective study on native Alaskan people was 27% [32], and 23% in a 23-year follow-up study in China [33]. However, this rate in our earlier cross-sectional study on young adults vaccinated in infancy in Mazandaran Province was 19% [34]. The mechanism of natural boosting was uncertain. However, it may result from a cross antigen reaction or from a transient HB viremia with no other evidence of HB infection. This phenomenon possibly leads to maintaining long-term immunity and memory against HB infection [35].

In this study, 0.78 and 4.6% of studied subjects were HBsAg and anti-HBc positive. These rates were not significantly different among those who were fully or incompletely vaccinated and also there was no correlation with their work’s experience. The frequency of HB infection in HCPs depends on the HBsAg prevalence in the general population where they work and the frequency of exposure to B/BF in their career [6–11]. Therefore, the reported prevalence varied considerably among HCPs working in different countries, and even within the same country. More than two decades after successful implementation of the universal infantile HB immunization along with HB vaccination of the high-risk groups and adolescents catch-up immunization program in the world and Iran, the prevalence of HB infection and HBsAg carrier reduced significantly among general population and some high-risk groups including HCP in the many countries and even within the same country in the world [2, 36]: 0.3% (0.15–2.7) in USA [37], 0.9% (0.1–4.4%) in EU [38], 1% in India [24], 4.7% in Indonesia [39], and 8.7% (5.2–14.3%) in Cameron [26]. The prevalence of HB infection in Iran reduced in line with its global downward trend [40, 41]. In this regard, the national prevalence of HBsAg and anti-HBc positive cases was estimated to be, respectively, 1.84% (95%CI: 1.61–2.09) and 13.59% (95%CI:12.92–14.29) in the range of 0.76–5.10% and 4.17–36.9% [40]. The frequency rates of HBsAg and anti-HBc in this study were lower than those reported in Iranian general population. It was similar to our recent findings from other teaching hospitals in Mazandaran Province, and some other parts of the country [42, 43]. However, the prevalence of HBsAg positivity in this study was higher than that the recent estimate among Iranian HCP: 0.4% (95%: 0.1–0.5) in the range of 0.3 and 4.1% [44]. Results showed that 4.6% of screened vaccinated personnel were anti-HBc positive, which in the majority of cases was associated with protective titers of ant-HBs antibody suggestive for resolved subclinical (breakthrough) HB infection. The frequency of positive rate was not significantly different among different profession. Symptomatic HB infection or HBsAg carrier status, following a successful HB immunization of HCP, was rare [6–8]. However, the reported prevalence of subclinical breakthrough HB infection in fully vaccinated HCP varied widely in the world. For example, the incidence rates were, respectively, 2.5, 2.5, and 2.5% in the USA [45], Spain [46] and Japan [47] versus 16.4% in Poland [20], 24.7% in India [24], and 70% in Albania [48]. The relatively low prevalence of HB infection reported in this and some other domestic studies could be attributed to the high rate of vaccination against HB among Iranian HCP [22, 23]. However, the role of the National Infants HB Immunization Program launched in 1993 in reducing the prevalence of HBsAg among Iranian population should not be ignored [40, 41].

According to our findings, approximately 12% of the vaccinated persons were serologically susceptible to HB infection. Three to 6 weeks after administering one dose of HB vaccine, 91% of boosted subjects responded and seroconverted, indicating the presence of long-lasting protective immune memory from the vaccination.

None of the international guidelines for HB immunization has recommended a routine administration of booster doses of HB vaccine or periodic anti-HBs antibody testing once the response to a full vaccination occurred in general, but special consideration for high-risk groups may be worthwhile [49]. HCP are regarded as a high-risk group to HB infection. Therefore, booster vaccination is strongly recommended for non-immune HCP, regardless of endemicity in the area concerned [6–8, 14, 16, 17, 49]. A recent Cochrane review was not able to identify any randomized study to assess the benefit of booster vaccination in preventing HB infection [50]. However, periodic anti-HBs testing is recommended in some European countries [18]. Some authorities [51, 52] also recommended booster injection to serologically susceptible persons [19, 24, 51, 52]. Our findings support the idea that no booster of HB vaccine is required after a successful primary vaccination. These findings are consistent with data published recently. The result from a long-term study, 10–31 years after the initial HB immunization on HCP, indicated that none of the participants was HBsAg positive, 2.5% of them showed evidence of resolved HB infection during their work’s experience, and approximately 23% of them had lost their seroprotection. After boosting, more than 94% of serosuseptible participants were seroconverted. Researchers concluded that HB vaccine provides long-term protection and thus booster vaccination is not necessary [45]. However, further studies in this area are still required. Nevertheless, highly infectious HB virus inoculum might overpower low/non-protective anti-HBs titers during the long professional life of HCP. In these situations, the administration of a booster dose of HB vaccine could be considered [6–8, 16, 17, 45, 51, 52].

This study showed that 0.91% of HCP were anti-HCV antibody positive. However, none of the PCR tested subjects were positive. This may be due to clearance of HCV infection by somebody, EIA test or HCV PCR were falsely reactive, and rarely that a person has intermittent or low level viremia. The prevalence of HCV infection varied markedly among different countries in the world. While central and East Asia, North Africa and the Middle East estimated to have high prevalence of infection rates (> 3.5%), most EU countries and Australia showed the prevalence of 1.5–3.5% and some other countries, including the most American countries, had the prevalence of less than 1.5%. During recent years, an upward trend of HCV infection was observed in the world [4] and in Iran [53]. However, the prevalence of HCV infection in Iran seems to be as low as 0.3–0.5% among blood donors [54, 55]. In this study, the prevalence of anti-HCV antibody positivity was higher than those Iranian blood donors. Although, the risk of HCV infection, following occupational exposure, was not high [10, 11, 16], nearly 1% of the prevalence rate found in this study indicated the necessity of appropriate post-exposure management in the cases of exposure to possible infectious sources. In this situation, regular follow-up evaluations are needed to diagnose possible HCV infection for early treatment [9–11]. However, because of low rate of HCV infection among the general population in Iran, pre-exposure screening of HCPs for HCV infection is not recommended.

For this study some limitations did exist. The most potential limitation was its reliance on self-reported data, which might influence the reliability of the findings and cause probable recall bias. Other limitation was that the HBV and HCV infection status before employment and the immune response to the initial HB vaccination series was not known. Moreover, the relation between the infection status and the NSIs was not investigated. Finally, for this study only hospital HCPs was investigated and health care providers in the primary health care centers and private clinics were not included, and this may influence the data collected for this group of personnel.

## Conclusion

Based on the study findings, appropriate policies should be made to measure HB immunity before recruitment. Since high-rate seroprotection and long-term immune memory observed in this study, a booster dose of HB vaccine does not appear to be required. HCV infection screening before recruitment does not seem necessary. However, in the cases of exposure to highly susceptible HBV or HCV sources, as was recommended by experts and authorities for appropriate management, immediate anti-HBs and/or anti-HCV antibodies serologic testing is recommended [6–13].

## Data Availability

Obtained for this study will be available from the corresponding author at a reason all request.

## Abbreviations

HBV: Hepatitis B Virus
HCV: Hepatitis C virus
HCP: Health Care Personnel
PVST: Post vaccination serological testing
WHO: World health organization
EU: European countries
HBig: Hepatitis B immunoglobulin
MAZUMS: Mazandaran University of Medical Sciences
TUMS: Tehran University of Medical Sciences
ELISA: Enzyme immune assay
IU/L: International unit/ liter
PCR: Polymerase Chain Reaction
IVDU: Intravenous Drug Users
